# Supplementary material for: Stress-Activated Protein Kinase Signalling Regulates Mycoparasitic Hyphal-Hyphal Interactions in Trichoderma atroviride
Source: J Fungi (Basel). 2021 May 6;7(5):365. doi: 10.3390/jof7050365 (PMC8148604; doi:10.3390/jof7050365)
Supplement: Supplementary file 1 [file jof-07-00365-s001.zip › jof-1210169 suppl.pdf]

Stress-activated protein kinase signalling regulates mycoparasitic hyphal-hyphal interactions in *Trichoderma atroviride.*

Dubraska Moreno-Ruiz[1], Linda Salzmann[1,‡], Mark D. Fricker[2,‡], Susanne Zeilinger[1] and Alexander Lichius[1,*]

[1]Department of Microbiology, University of Innsbruck, 6020 Innsbruck, Austria; dubraska.moreno-ruiz@uibk.ac.at (D.M-R.), Susanne.Zeilinger@uibk.ac.at (S.Z.), linda.salzmann@risch.ch (L.S.)

[2] Department of Plant Sciences, University of Oxford, Oxford, OX1 3RB, United Kingdom. mark.fricker@plants.ox.ac.uk (M.D.F.)

* Correspondence: alexander.lichius@uibk.ac.at

Supplementary data:

Table S1. Plasmids used and constructed within this study

Table S2 Primers used for *T. atroviride* CRIB reporter vector construction

Table S3. Primers used for generation of *tmk1*-CRIB and *tmk3*-CRIB gene deletion cassettes and genotyping.

Table S4. Fluorescent strains used in this study

Figure S1. Construction of a CRIB reporter based on the PAK Cla4 of *T. atroviride* and the improved mbasic GFP.

Figure S2. Pharmacological inhibition of GTPase activity.

Figure S3. Panoramic view of confrontation between *T. atroviride* (CRIB-GFP) and *B. cinerea* (cytoplasmic DsRed) displaying the extent of prey-induced depolarisation evident by CRIB reporter dislocation and dispersal.

Figure S4. Antagonistic features of *T. atroviride* MAPK mutants in confrontations against *B. cinerea* and *F. oxysporum* on PDA.

Figure S5. Observation frequencies of contact-induced hyphal growth phenotypes of *T. atroviride* in interaction with either *B. cinerea* (Bc) or *F. oxysporum* (Fo).

Figure S6. *T. atroviride* wild type and MAPK mutants show phenotypic hyphal avoidance while in pre-contact phase.

Figure S7. Cellular damage induced by pre-contact interaction with *B. cinerea* in *T. atroviride* Δ*tmk3*-CRIB hyphae.

Video S1. Functional localization dynamics of the CRIB reporter in the *T. atroviride* wildtype.

Video S2. Lateral displacement of GTPase activity clusters at the tip apex during hyphal self-avoidance responses in *T. atroviride* wildtype.

Video S3. CRIB reporter dispersal and concomitant tip growth arrest in *T. atroviride* wildtype during pre-contact with *B. cinerea*.

Video S4. Wavy growth pattern of *T. atroviride* WT-CRIB in contact to *B. cinerea* hyphae.

Video S5. Contact-induced multi-polarisation of *T. atroviride* WT-CRIB hyphae upon contact with *R. microsporus* hyphae.

Video S6. Contact-induced multi-polarisation of *T. atroviride* WT-CRIB hyphae upon contact with *R. microsporus* hyphae.

Video S7. Chemotropic avoidance of *T. atroviride* Δ*tmk1*-CRIB in close proximity to hyphae of *F. oxysporum* during contact phase.

Video S8. Chemotropic attraction of *T. atroviride* Δ*tmk3*-CRIB towards hyphae of *F. oxysporum* upon contact phase.

Video S9. Chemotropic attraction of *T. atroviride* WT-CRIB hyphae towards hyphae of *F. oxysporum* upon contact phase.

Video S10. Chemotropic attraction of *T. atroviride* Δ*tmk3*-CRIB hyphae towards hyphae of *F. oxysporum* upon contact phase.

Video S11. Chemotropic attraction of *T. atroviride* Δ*tmk3*-CRIB hyphae displaying multi-polarization, and hyphal death during contact phase in confrontation with *B. cinerea.*

Table S1. Plasmids used and produced in this study

Plasmid	Description	Source
pZEGA1	transformation vector without selection marker, originally for co-transformation with pHATα (P*pki:: sgfp*)	(Zeilinger et al. 1999)
pGFP-XYR1	Plasmid containing the *hph* resistence gene	(Lichius et al., 2014)
pLS1	transformation vector for cytoplasmic sGFP expression (P*pki::sgfp*) based on pZEGA1 backbone	This study
pLS1-CRIBc	expression vector for CRIBc-sGFP fusion construct encoding amino acids 55-263 from *T. atroviride* p21-activated kinase (PAK) Cla4 linked to sGFP (P*pki::crib*$_{cla4}$*-sgfp*)	This study
pLS2	transformation vector for cytoplasmic mBasicGFP expression (P*pki::basicgfp*) based on pLS1	This study
pLS2-CRIBc	expression vector for CRIBc-mBasicGFP fusion construct encoding amino acids 55-263 from *T. atroviride* p21-activated kinase (PAK) Cla4 linked to basicGFP (P*pki::crib*$_{cla4}$*-mBasicGFP*)	This study
pLS3	transformation vector for cytoplasmic mBasicGFP expression (P*pki::mBasicGFP*) based on pLS2	This study
pLS3-CRIBc	expression vector for CRIBc-mBasicGFP fusion construct encoding amino acids 55-263 from *T. atroviride* p21-activated kinase (PAK) Cla4 linked to mBasicGFP (P*pki::crib*$_{cla4}$*- mBasicGFP*)	This study

Table S2. Primers used for *T. atroviride* CRIB reporter vector construction

Primer name	5'-3' sequence
pZEGA-lin-F	AGCTTGGCGTAATCATGG
pZEGA-lin-R	TGTGCTGCGGAATCATTA
Pgpdh-IF-F	ATGATTCCGCAGCACA**AGCTT**GAGAGCTACCTTACATCAA
Tgpdh-IF-R	GACCATGATTACGCCAAGCTTGGTACTATGGCTTAGATGG
GFP-F	ATGGTGAGCAAGGGCGA
pZEGA-MCS-R	GGGATCGAATTCCTGCAG
CRIB-Cla4-IF-F	GGCTGCAGGAATTCGATCCC**ATG**ACATATACCGGTTCCACG
CRIB-Cla4-IF-R	TCCTCGCCCTTGCTCACCAT**GCCTCCGCCTCCGCC**GGCAAAGGGGTCATCA

SDM-primers with oligonucleotide mismatches (bold).

Primer name	5'-3' sequence
sGFP-QC-F	CGTGACCACC**C**T**G**ACCTACGGCGTGCAGTGCTTC**GC**CCGCTACCCCG
sGFP-QC-R	CGGGGTAGCGG**GC**GAAGCACTGCACGCCGTAGGT**C**A**G**GGTGGTCACG
basicGFP-QC-F	CCTGAGCACCCAGTCC**AAG**CTGAGCAAAGACCCCA
basicGFP-QC-R	TGGGGTCTTTGCTCAG**CTT**GGACTGGGTGCTCAGG

Primers used for verification of correct cloning

Primer name	5'-3' sequence
Tcbh2-F	TCCACCTCTTGCGCTTCTCC
ORI-R	GAAGGGAGAAAGGCGGACA
Ppki2-F	CTGATTTCTCTCCCCCCCTC
sGFP-R	TTACTTGTACAGCTCGTCCATG
GFP-F	ATGGTGAGCAAGGGCGA
hphR-R	CCACGCCCTCCTACATCGAA
Tcbh-F	TCCACCTCTTGCGCTTCTCC
ORI-R	GAAGGGAGAAAGGCGGACA

Table S3. Primers used to generate Δ*tmk1*-CRIB and Δ*tmk3*-CRIB gene deletion cassettes and for PCR genotyping.

Oligonucleotide	5′- 3′ Sequence
	**For Δ*tmk1*-CRIB Plasmid Construction**
BbpDM-nat1-Fw	GTGCTGTGTTCCTCAGAATGG
BbpDM-nat1-Rv	GTTAACATGACCAGACTGATCGC
nat1- -Fw	CAGTCTGGTCATGTTAACCAACTGATATTGAAGGAGCATTTTTTGG
Nat1-Rv	GGCCCATTCTGAGGAACACAGCACTCAGGGGCAGGGCATGC
	**For Δ*tmk3*-CRIB Plasmid Construction**
BbpDM-tmk3-CRIB-Fw	GATAACGGTGAGACTAGCGG
BbpDM-tmk3-CRIB-Rv	TATTCCTTTGCCCTCGGAC
5′ Ptmk3-Fw	AGGCGATTAAGTTCTGGATGATTGGAGGGGG
5′ Ptmk3-Rv	ACCGGCCGCTAGTCTCACCGTTATCATTCAGATTTCGATTTTTTGGG
3′ Ptmk3-Fw	GCACTCGTCCGAGGGCAAAGGAATAGCGCCCGCGCACGCACGA
3′ Ptmk3-Rv	ATTACGCCAAGCTTGGTGGCCACCCATTGCGCATG
	**Fragment Amplification for Transformation**
pLS-trafo-F	AACTGTTGGGAAGGGCGA
pLS-trafo-R	TGTGTGGAATTGTGAGCGG
	***PCR* Genotyping**
Geno_hphR-Fw	GAGCTGATGCTTTGGGCC
Geno_3′ Ttmk3- Rv	TCTCTTGATTTCTACACAAACAGGG
Geno_5′ Ptmk3-Fw(2)	CCCTGAAGCTGCTGGATGGA
Geno_CRIB-mid-Rv	CCTCCGTCTTGTGAAAGTCG
Geno_Tmk3-Fw	ACTCAGACCTCCAGCCTGTG
Geno_Tmk3-R	ATAACATCGTCCGGGGGCG
Actin-T.a-Fw	CTACGGACCAGCAATACGGG
Actin-T.a-Rv	CATACAGGATCGCAAGATCTGC
Geno_nat-1-Fw	GGATGGGTCCTTCACCACC
Geno_Tgapdh-Rv	CCCCAGTCCAGATCATGATTGG

Table S4. Transformant strains generated in this study

Strain	Genotype
**WT-CRIB 1 and 2**	*Ppki::CRIBc-mbasicgfp::Tcbh2*<br>*T. atroviride* wild type strain P1 containing expression vector for CRIBc-mBasicGFP and Hygromycin resistance cassette
**Δ*tmk1*-CRIB 1 and 2**	*Ppki::CRIBc-mbasicgfp::Tcbh2*<br>◎*tmk1* MAPK deletion mutant of *T. atroviride* P1 (Reithner et al. 2007), containing expression vector for CRIBc-mBasicGFP and Nourseothricin resistance cassette
**Δ*tmk3*-CRIB 1 and 2**	*Ptmk3::CRIBc-mbasicgfp::Ttmk3*<br>◎*tmk3* SAPK deletion mutant of *T. atroviride* P1 (Moreno-Ruiz et al. 2020), containing expression vector for CRIBc-mBasicGFP and Hygromycin B resistance cassette

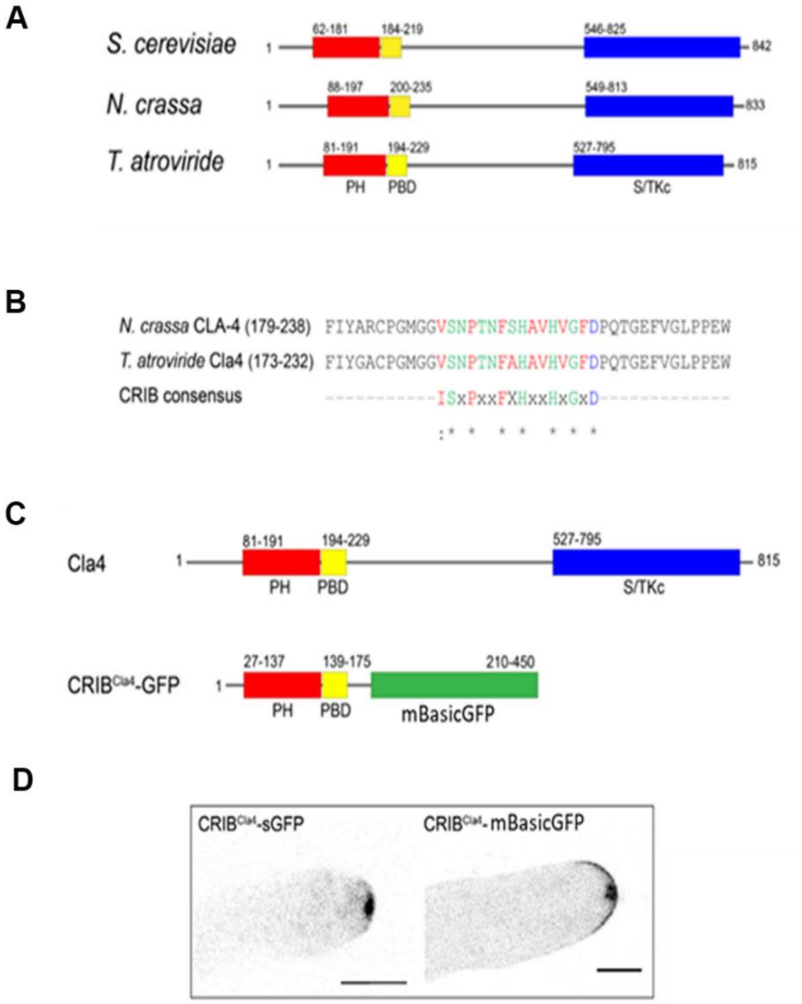

Figure S1. Construction of a CRIB reporter based on the PBD of the *T. atroviride* PAK Cla4 and the photophysically improved sGFP variant mBasicGFP. (A) The Cdc42-activated signal transducing kinase Cla4 is a member of the p21-activated kinase (PAK) family and is highly conserved amongst fungi. Cla4 orthologs of *Saccharomyces cerevisiae* (YNL29W), *Neurospora crassa* (NCU00406.2) and *T. atroviride* (Triat2.316062) all contain a pleckstrin homology (PH) domain essential for direct plasma membrane binding and functional localization of the PAK (Takahashi and Pryciak, 2007), the CRIB motif-harbouring p21-binding domain (PBD), and the catalytic Serine, Threonine Kinase (S/TKc) domain. **(B)** The CRIB domain of *T. atroviride* Cla4 was identified by alignment against CLA-4 of *N. crassa* (Lichius et al., 2014) and the eukaryotic CRIB consensus sequence (Bishop and Hall, 2000). The homology between the *T. atroviride* and *N. crassa* CRIB domain is 93.75%. **(C)** The *T. atroviride* CRIB reporter was constructed by fusing the N-terminal part of Cla4 comprising the PH and PBD domains to mBasicGFP resulting in the CRIB^{Cla4}-mBasicGFP fusion construct. MBasicGFP is a GFP variant generated from sGFP(S65T) (Heim et al. 1995; Zeilinger et al. 1999) by introducing a number of amino acid substitutions (F64L/S65T/S72A/I167T/A206K) that confer enhanced fluorescent brightness and monomerisation compared to the initial CRIB^{Cla4}-sGFP,overall providing considerably improved visibility, better signal-to-noise ratio and higher spatial resolution of activated GTPases at hyphal tips where they typically localise as apical cap and part of the Spitzenkörper. Scale bar, 5 µm.

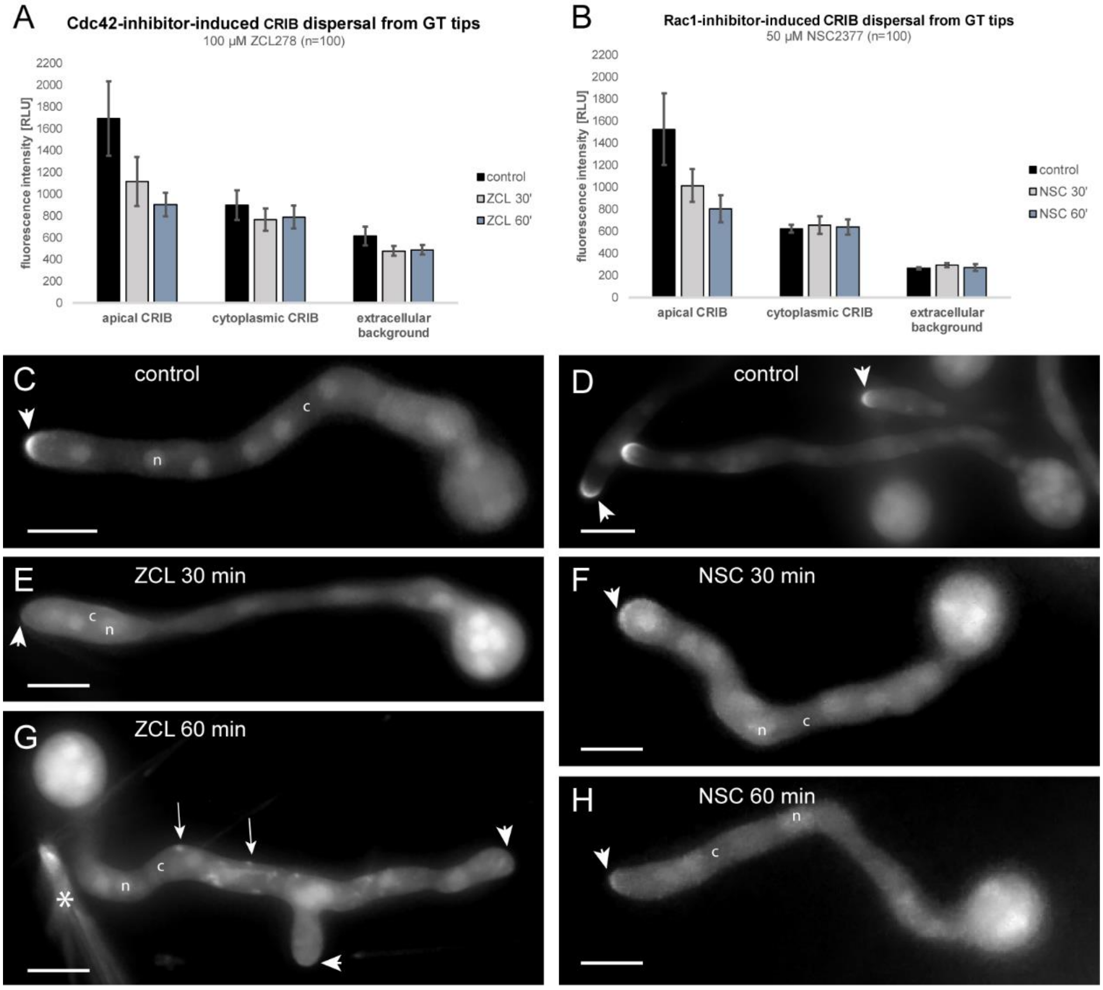

Figure S2. Pharmacological inhibition of GTPase activity. (A) 30-minute treatment with 100 µM of the Cdc42-GEF-specific inhibitor ZCL278 reduces the apical CRIB signal to 66% of that of the untreated control, confirming rapid dispersal of Cdc42-activity clusters from the tip apex. After 60 minutes exposure to the drug, apical fluorescence is down to 54% and levelled with the cytoplasmic CRIB signal. Dispersal of apical GTPase activity coincides with tip growth arrest in all instances. **(B)** 30-minute treatment with 50 µM of the Rac1-GEF-specific inhibitor NSC23766 follows very similar pharmacokinetics, however, does never lead to the complete removal of apical CRIB reporter fluorescence. **(C&D)** In the untreated controls, intense CRIB reporter fluorescence localises Cdc42- and Rac1-activity clusters at the growing germ tube apices (arrowheads). Nuclear (n) CRIB reporter fluorescence is slightly elevated over the cytoplasmic (c) population. **(E)** 30-minute treatment with ZCL278 remove distinguishable CRIB reporter fluorescence from the tip apex, whereas **(F)** in the presence of NSC23766 apical CRIB reporter fluorescence is still visible albeit significantly reduced. This indicates that Cdc42 activity remains to drive germ tube tip growth to some extent. This is in line with our previous findings in *N. crassa* (Lichius et al. 2014). **(G)** After 60 minutes, small atypical clusters of CRIB reporter fluorescence appear (arrows) that might indicate drug artefacts. Notably, ZCL278 crystals (asterisk) precipitate in the medium, reducing bioavailability of the drug. **(H)** Apical CRIB reporter fluorescence and hence GTPase activity cluster formation is further reduced but still not completely inhibited after 60 minutes in the presence of NSC23766. Scale bars, 10 µm. Error bars represent standard deviation of 100 individual measurements for each condition.

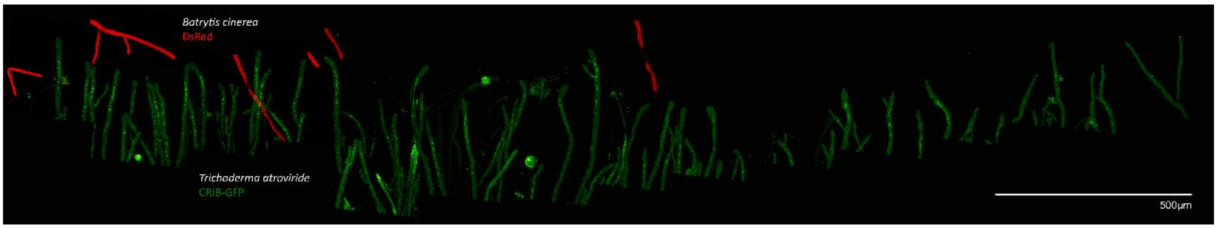

Figure S3. Panoramic view of confrontation between *T. atroviride* (CRIB-GFP) and *B. cinerea* (cytoplasmic DsRed) displaying the extent of prey-induced depolarisation evident by CRIB reporter dislocation and dispersal.

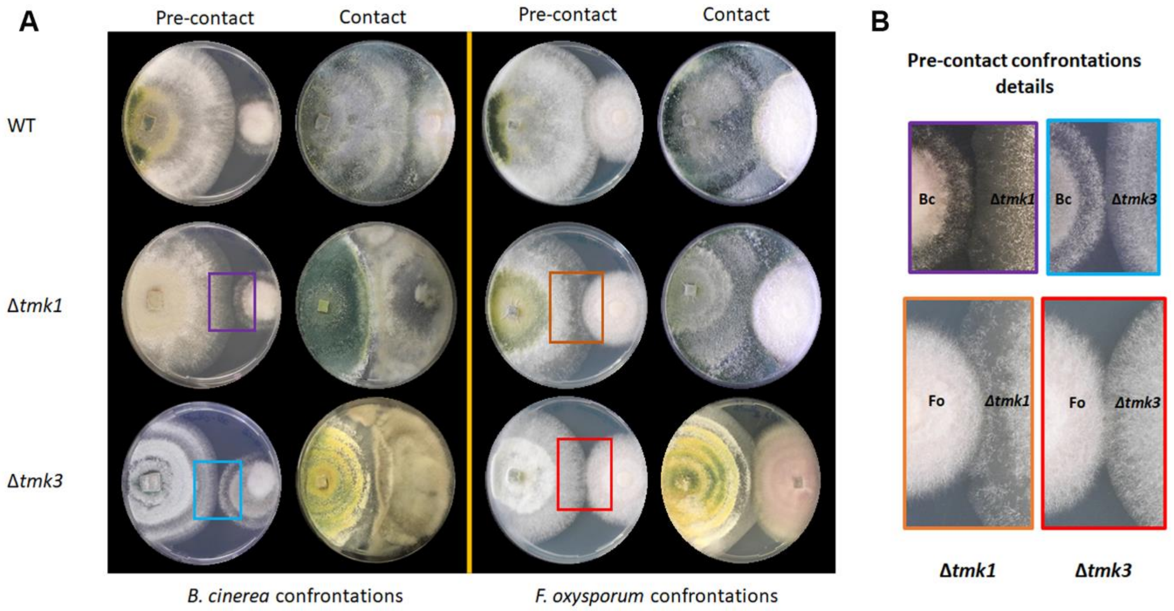

Figure S4. Pre-contact and contact features of *T. atroviride* MAPK mutants in confrontations against *B. cinerea* and *F. oxysporum* on PDA. (A) Dual confrontations with *B. cinerea* and *F. oxysporum* on PDA were incubated in the presence of 12h/12h light–dark cycles and evaluated daily for seven days. **(B)** Detail of colony margins during pre-contact phase during confrontations with fungal preys showing the earlier onset of gap formation in Δ*tmk1* compared to Δ*tmk3*. FP = fungal prey; M = mycoparasite.

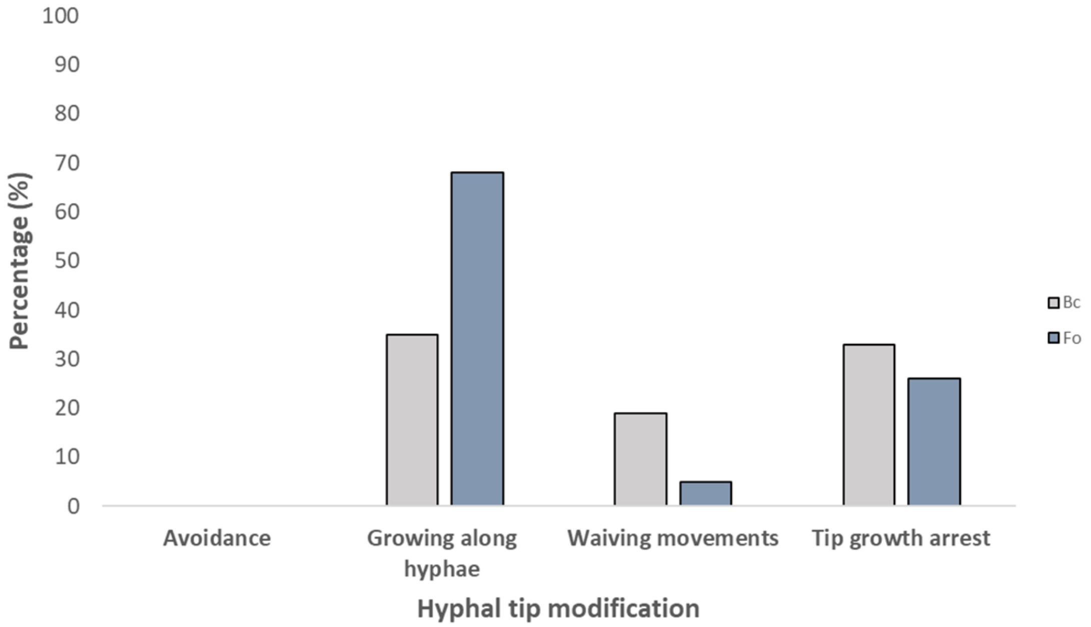

Figure S5. Observation frequencies of contact-induced hyphal growth phenotypes of *T. atroviride* in interaction with either *B. cinerea* (Bc) or *F. oxysporum* (Fo).

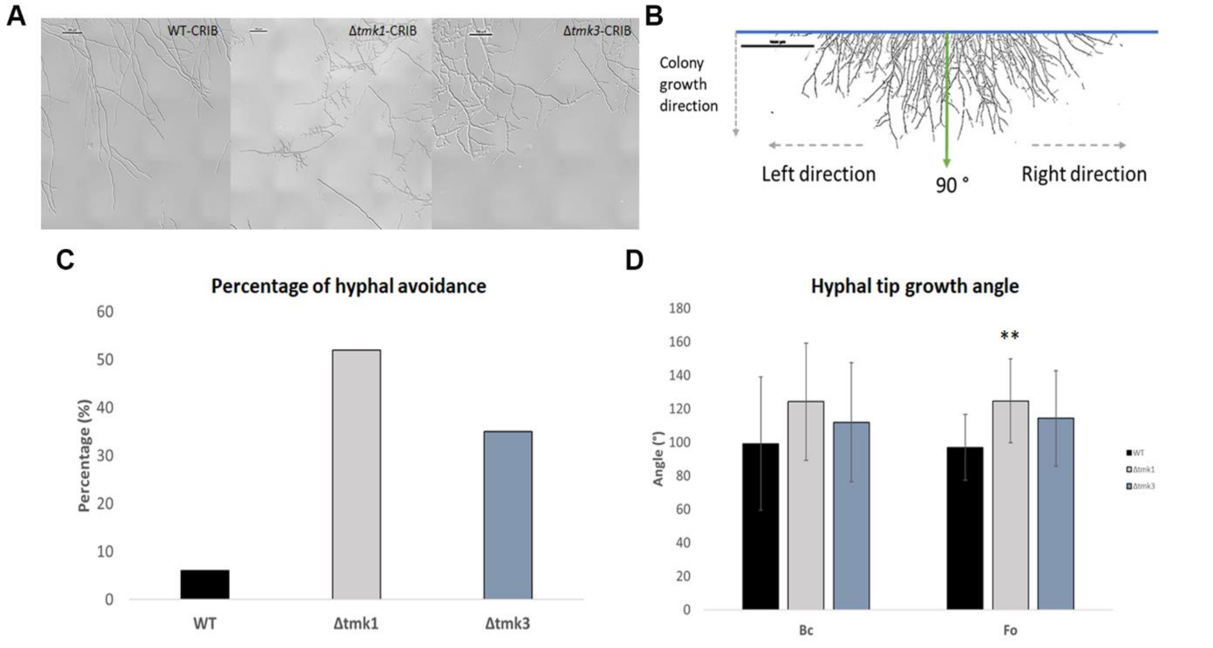

Figure S6. Quantification of avoidance response of *T. atroviride* wild type and MAPK mutants in the pre-contact phase. (A) Representative DIC images of the pre-contact phases in dual confrontations with *B. cinerea* on MM9. Scale bars, 100 µm. **(B)** Micrograph of the colony margin of *T. atroviride* in normal, solitary growth. Directional arrows along the confrontation plane were used to determine the prevailing hyphal growth direction relative to the corresponding 90° reference. Scale bar, 1 mm. **(C)** Quantification of the percentage of hyphal avoidance in confrontation assays between *T. atroviride* wild type and MAPK mutants against *B. cinerea* or *F. oxysporum*, respectively. Both MAPK mutants showed significantly stronger negative chemotropism in response to prey presence than the wild type. Number of experiments n=10. **(D)** The comparison of changes in hyphal tip growth angles indicates that Δ*tmk1*-CRIB has a slightly higher tendency to redirect its hypha in response to prey-derived signals than WT-CRIB and Δ*tmk3*-CRIB. Number of experiments n=10.

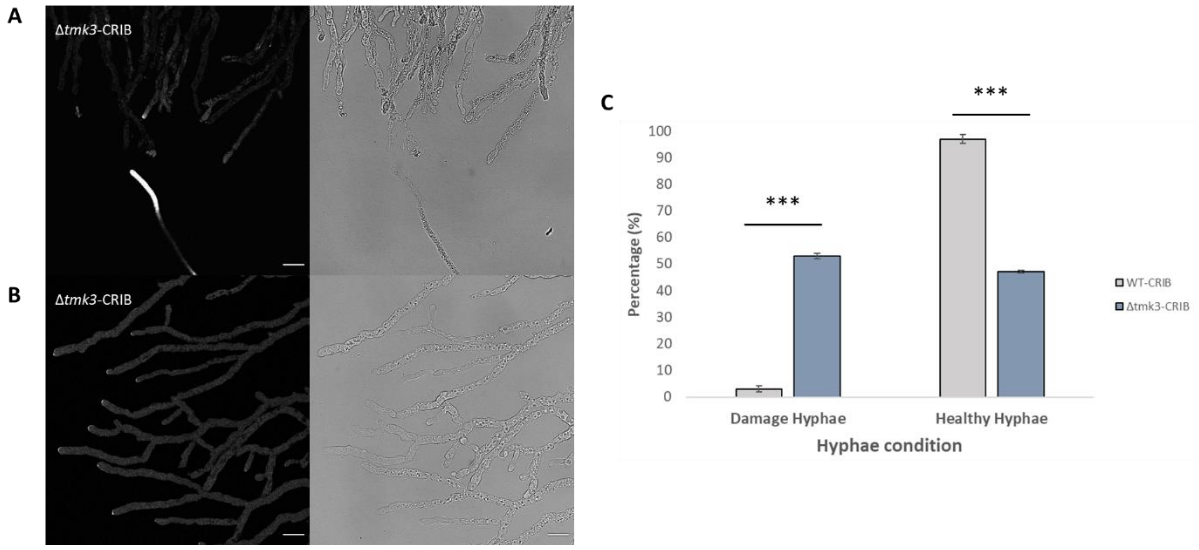

Figure S7. Cellular damage induced by pre-contact interaction with *B. cinerea* in *T. atroviride* Δ*tmk3*-CRIB hyphae. (A) Leading hyphae of *T. atroviride* Δ*tmk3*-CRIB suffered from cell lysis and impaired polarised growth when confronted with compounds released by *B. cinerea* into the culture medium. Scale bar, 20 μm. **(B)** Hyphae growing in an axenic culture displaying normal growth morphology and functional recruitment of the CRIB reporter. Scale bar, 20 μm. **(C)** Percentage of affected hyphae observed during pre-contact interaction with *B. cinerea* at a distance of 0.5-1 mm. Number of experiments n=10.

References:

Heim, Roger; Cubitt, Andrew B.; Tsien, Roger Y. (1995): Improved green fluorescence. In *Nature* 373 (6516), pp. 663–664.

Lichius, Alexander; Goryachev, Andrew B.; Fricker, Mark D.; Obara, Boguslaw; Castro-Longoria, Ernestina; Read, Nick D. (2014): CDC-42 and RAC-1 regulate opposite chemotropisms in *Neurospora crassa*. In *J Cell Sci* 127 (Pt 9), pp. 1953–1965. DOI: 10.1242/jcs.141630.

Moreno-Ruiz, Dubraska; Fuchs, Alessandro; Missbach, Kristina; Schuhmacher, Rainer; Zeilinger, Susanne (2020): Influence of Different Light Regimes on the Mycoparasitic Activity and 6-Pentyl-α-pyrone Biosynthesis in Two Strains of Trichoderma atroviride. In *Pathogens (Basel, Switzerland)* 9 (10). DOI: 10.3390/pathogens9100860.

Reithner, Barbara; Schuhmacher, Rainer; Stoppacher, Norbert; Pucher, Marion; Brunner, Kurt; Zeilinger, Susanne (2007): Signaling via the *Trichoderma atroviride* mitogen-activated protein kinase Tmk 1 differentially affects mycoparasitism and plant protection. In *FG & B* 44 (11), pp. 1123–1133. DOI: 10.1016/j.fgb.2007.04.001.

Zeilinger, S.; Galhaup, C.; Payer, K.; Woo, S. L.; Mach, R. L.; Fekete, C. et al. (1999): Chitinase gene expression during mycoparasitic interaction of *Trichoderma harzianum* with its host. In *FG & B* 26 (2), pp. 131–140. DOI: 10.1006/fgbi.1998.1111.
