# Supplementary figures and images for: Stress-Activated Protein Kinase Signalling Regulates Mycoparasitic Hyphal-Hyphal Interactions in Trichoderma atroviride
Source: J Fungi (Basel). 2021 May 6;7(5):365. doi: 10.3390/jof7050365 (PMC8148604; doi:10.3390/jof7050365)

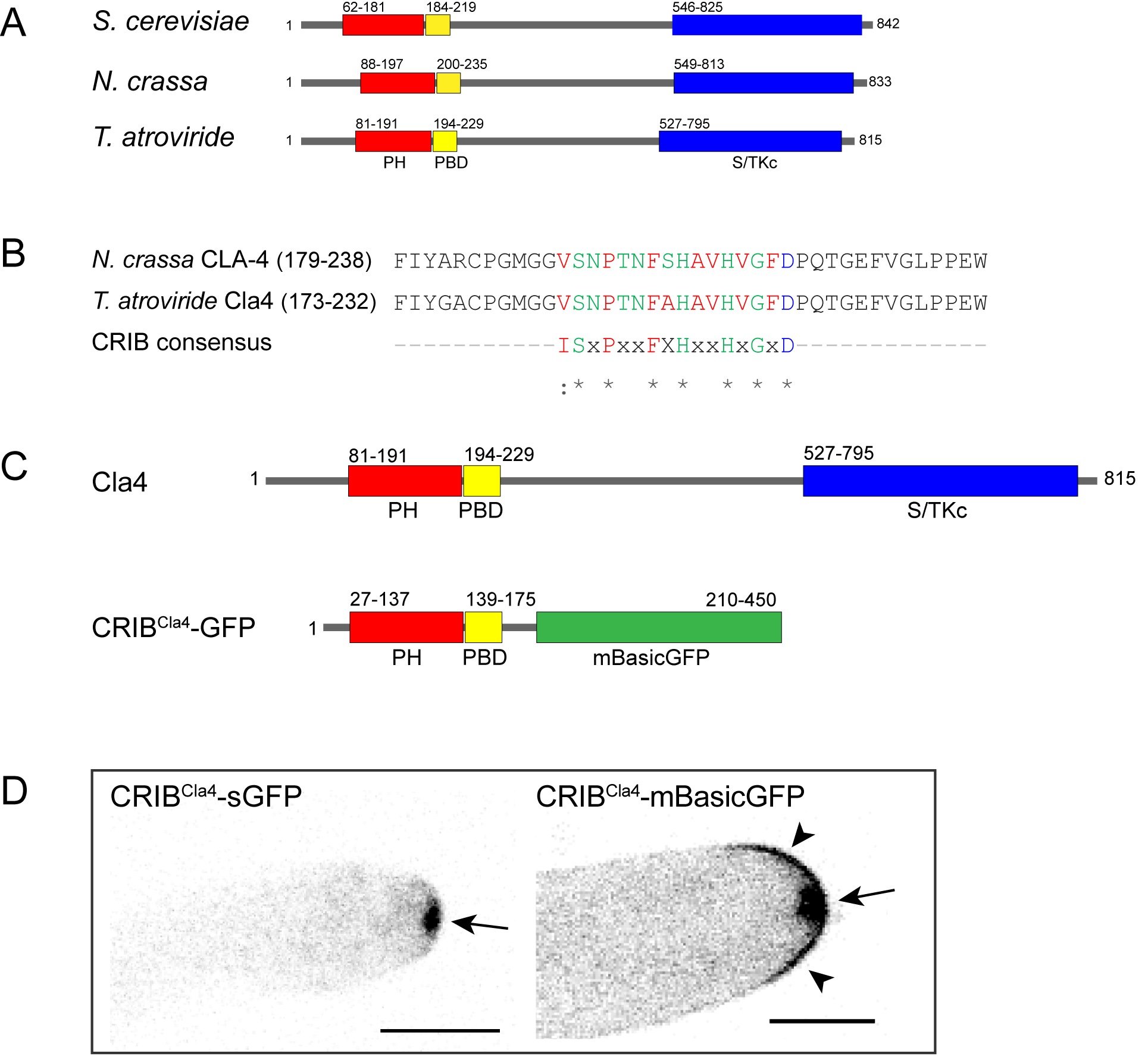

Supplement: Supplementary file 1 [file jof-07-00365-s001.zip › Fig S1_new_300dpi.tif]

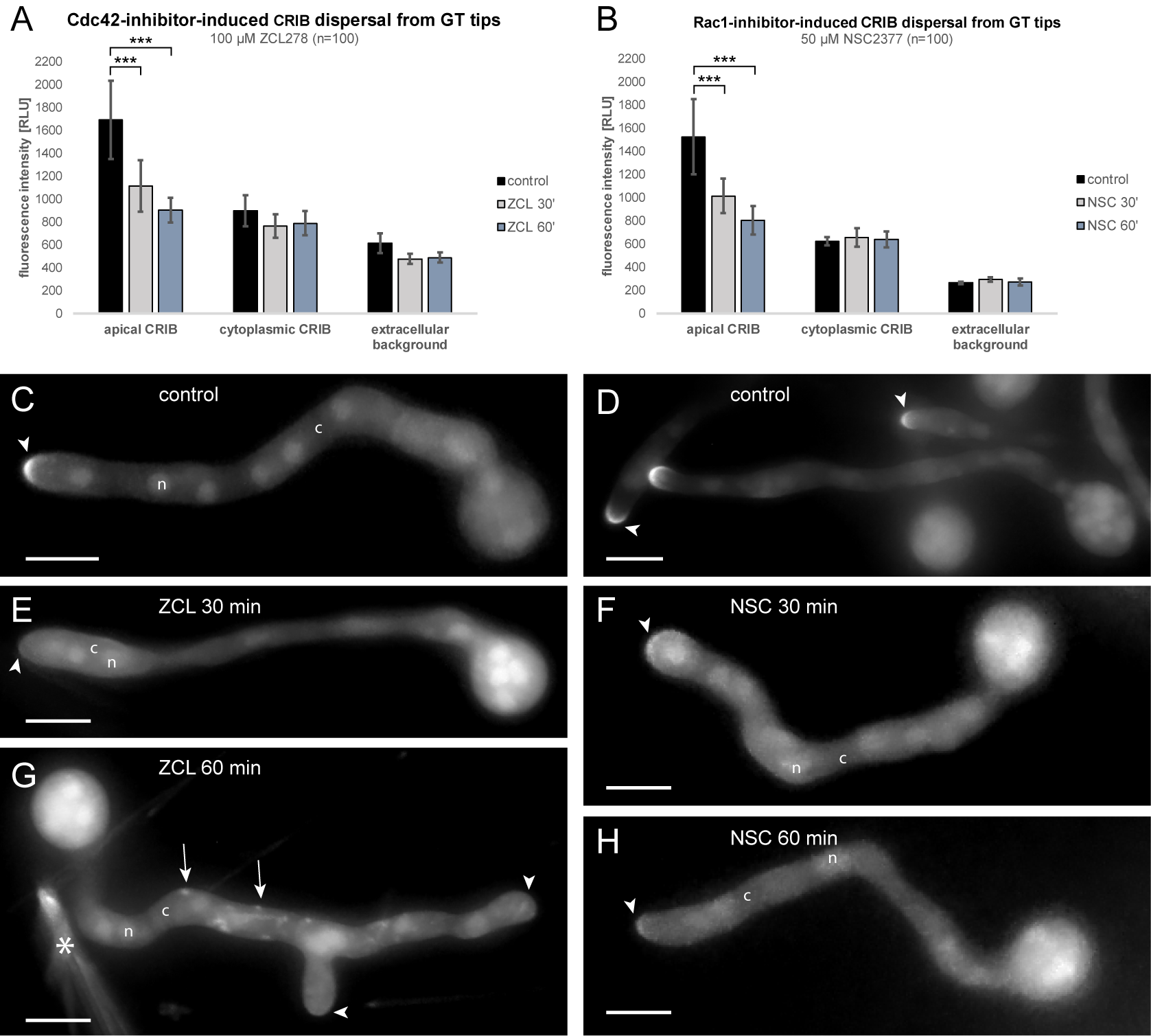

Supplement: Supplementary file 1 [file jof-07-00365-s001.zip › Fig S2_new_300dpi.tif]

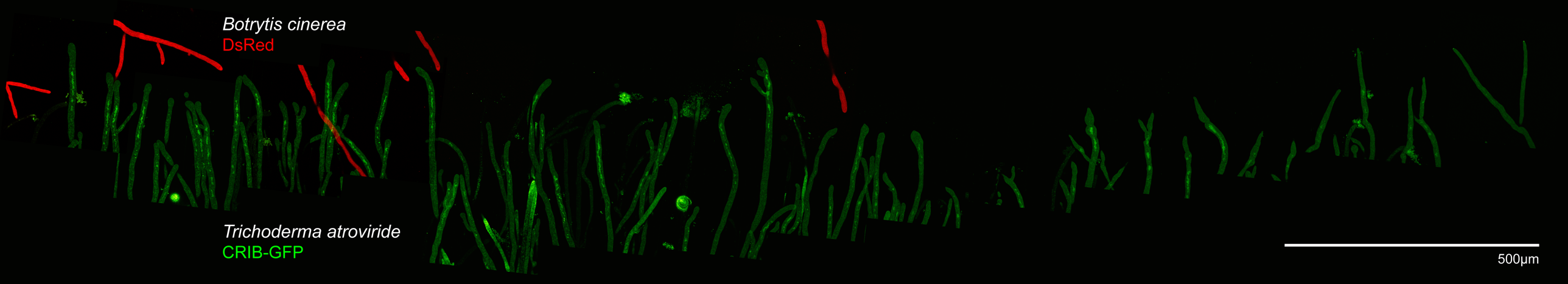

Supplement: Supplementary file 1 [file jof-07-00365-s001.zip › Fig S3_new_300dpi.tif]

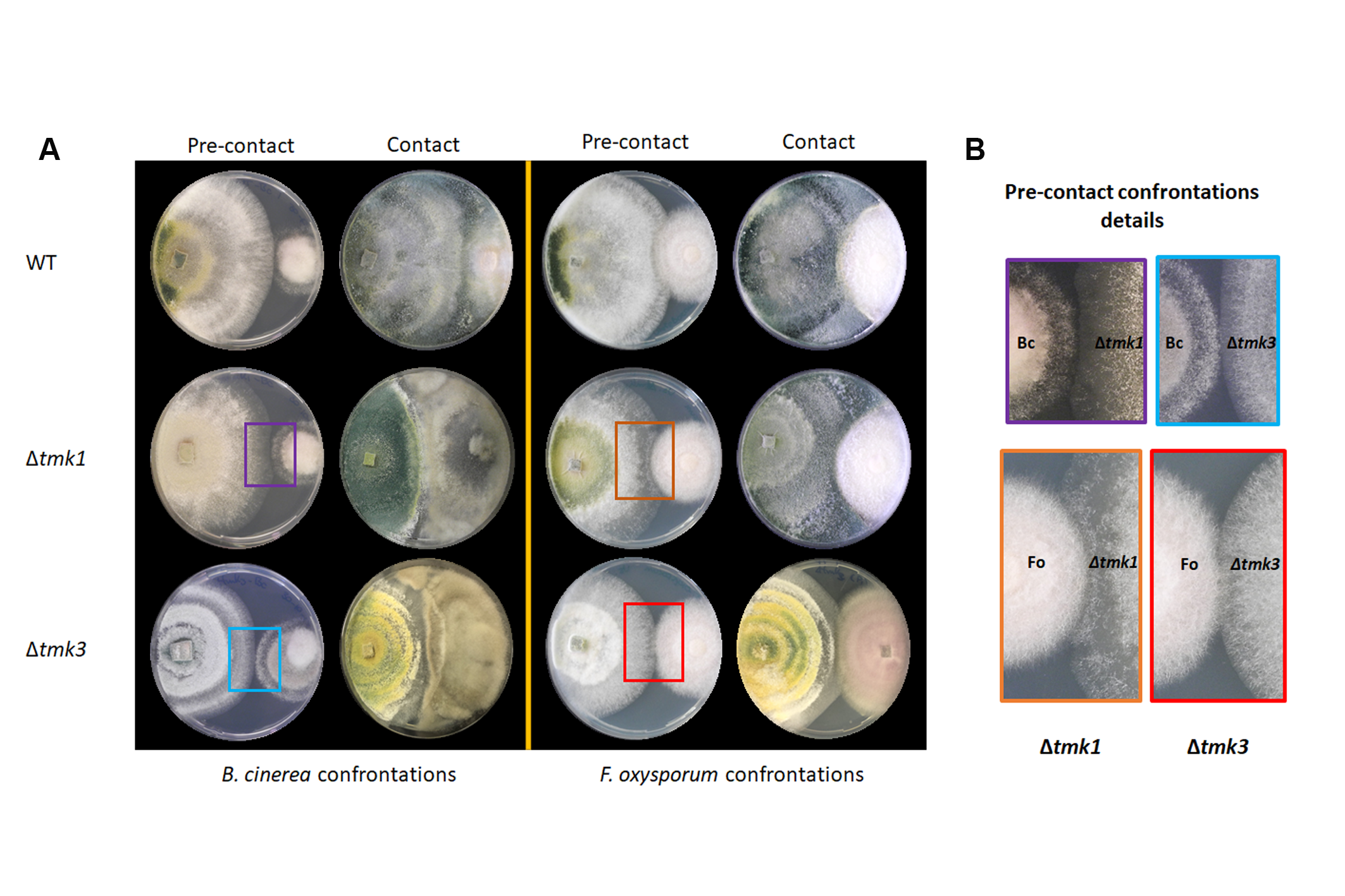

Supplement: Supplementary file 1 [file jof-07-00365-s001.zip › Fig S4_300dpi.tif]

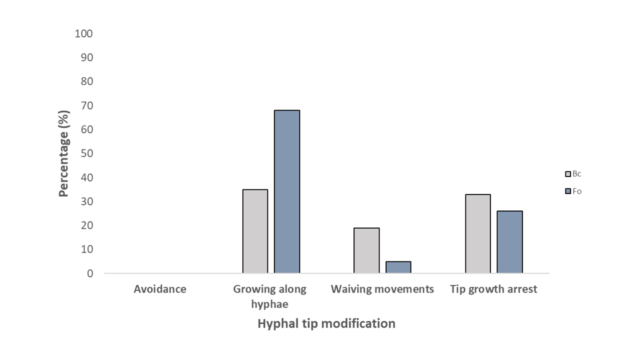

Supplement: Supplementary file 1 [file jof-07-00365-s001.zip › Fig S5_300dpi.tif]

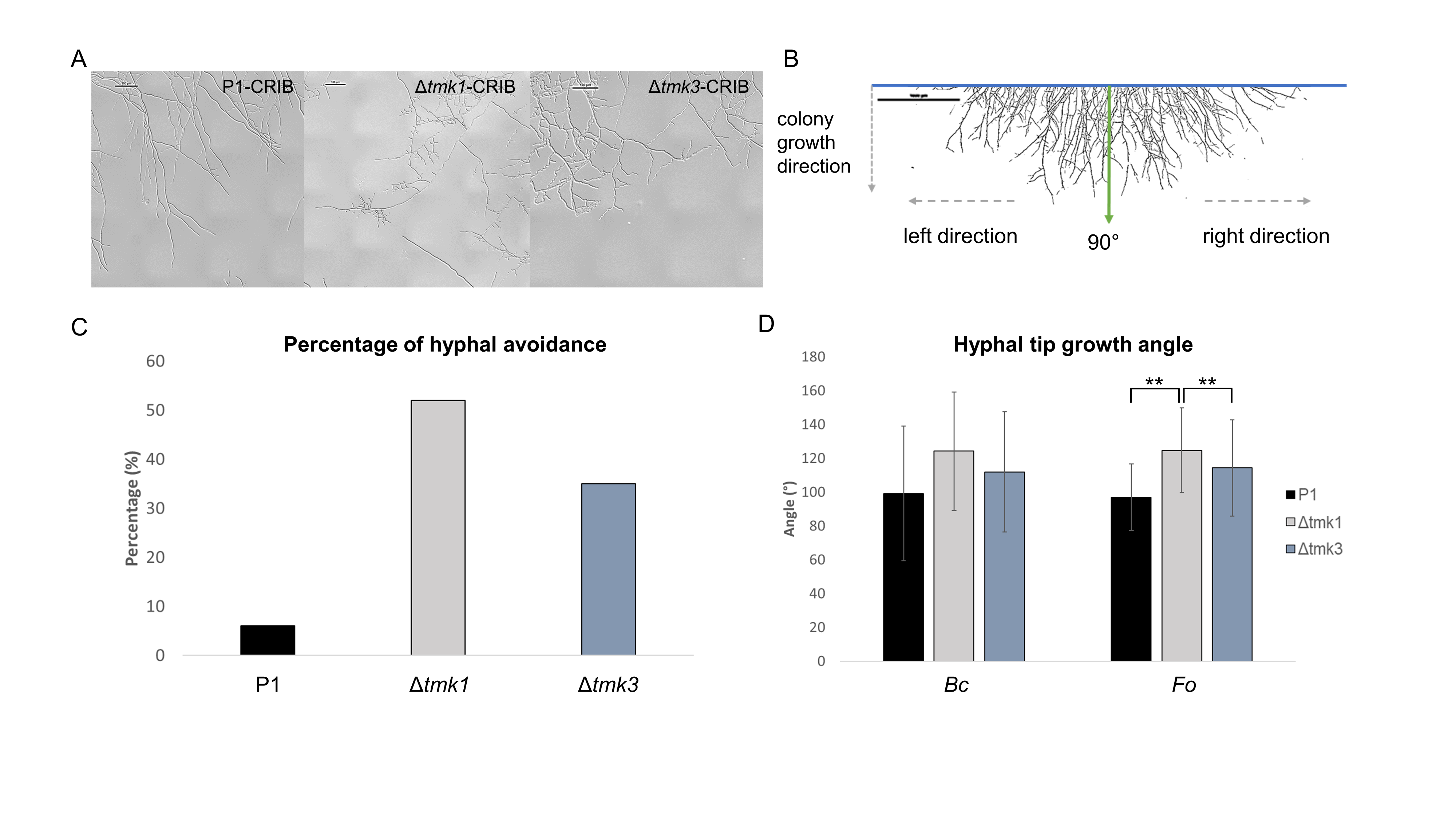

Supplement: Supplementary file 1 [file jof-07-00365-s001.zip › Fig S6_new_300dpi.tif]

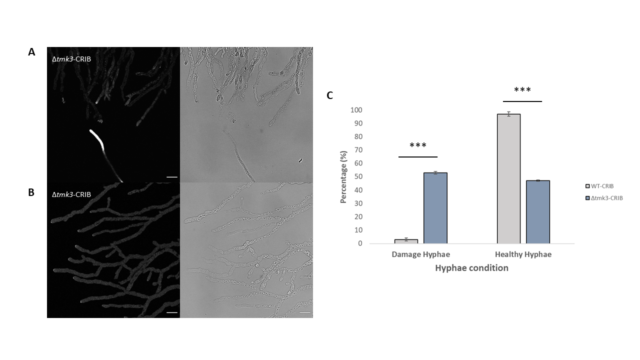

Supplement: Supplementary file 1 [file jof-07-00365-s001.zip › Figure_S7.tif]
